# Appendiceal Orifice Inflammation Is Associated with Lower Rate of Complete Endoscopic Remission in Patients with Ulcerative Colitis

**DOI:** 10.3390/jpm12050748

**Published:** 2022-05-05

**Authors:** Chang Kyo Oh, Han Hee Lee, Jin Su Kim, Bo-In Lee, Young-Seok Cho

**Affiliations:** 1 Division of Gastroenterology, Department of Internal Medicine, Kangnam Sacred Heart Hospital, Hallym University College of Medicine, Seoul 07441, Korea; ckdryekd@gmail.com; 2 Division of Gastroenterology, Department of Internal Medicine, Yeouido St. Mary’s Hospital, The Catholic University of Korea, Seoul 07345, Korea; hanyee99@hanmail.net; 3 Division of Gastroenterology, Department of Internal Medicine, Eunpyeong St. Mary’s Hospital, The Catholic University of Korea, Seoul 03312, Korea; jinsu23@naver.com; 4 Division of Gastroenterology, Department of Internal Medicine, Seoul St. Mary’s Hospital, The Catholic University of Korea, Seoul 06591, Korea; gidoc4u@gmail.com

**Keywords:** appendiceal orifice inflammation, endoscopic remission, ulcerative colitis

## Abstract

Appendiceal orifice inflammation (AOI) is commonly considered a skip lesion in ulcerative colitis (UC). However, the clinical significance of AOI in UC patients remains controversial. This study aimed to evaluate the clinical feature and long-term outcomes of AOI by comparing UC patients with and without AOI. This study was conducted as a retrospective design of patients who were newly diagnosed or referred within 3 months after diagnosis at Seoul St. Mary’s Hospital from 1 January 2001 to 31 December 2020. All patients underwent index and follow-up colonoscopies. The long-term outcomes involved achieving complete endoscopic remission (ER), use of biologics, hospitalization, and proximal disease extension. Complete ER was defined as Mayo endoscopic subscore 0. In total, 318 UC patients were included, of which 140 had AOI. The baseline characteristics were not significantly different between AOI and non-AOI groups. The cumulative risk of complete ER was a significant difference between AOI and non-AOI groups (*p* = 0.041). The other cumulative risks of disease outcomes were not significantly different between AOI and non-AOI groups (use of biologics, *p* = 0.542; hospitalization, *p* = 0.795; proximal disease extension, *p* = 0.403). The multivariate Cox regression analysis also revealed that AOI was the significant factor of complete ER (hazard ratio, 0.656; 95% confidence interval, 0.462–0.932; *p* = 0.019) in UC patients. AOI shows a significant association with lower rate of complete ER in UC patients. Therefore, a meticulous treatment strategy may be recommended to achieve complete ER in UC patients with AOI.

## 1. Introduction

Ulcerative colitis (UC) is a chronic inflammatory bowel disease characterized by abdominal pain, diarrhea, mucoid stool, and rectal bleeding [1]. Its disease course is typically characterized by repeated asymptomatic remissions and symptomatic relapse. Most mucosal inflammation in UC typically originates in the rectum, extending to the proximal colon. Proximal extension has a poor disease course, including relapse, risk of colectomy, and hospitalization [2]. Previously, the treatment of UC aims to achieve clinical remission. Recently, endoscopic remission (ER) has emerged as a relevant treatment goal in UC because accumulated evidence suggested that it was associated with a favorable disease course, including prolonged clinical remission and a lower colectomy rate [3,4]. Appendiceal orifice inflammation (AOI) is often found as a skip lesion in UC patients [5,6]. Although there have been several studies on the clinical significance and prognostic implication of AOI [7,8,9,10,11,12,13], its clinical significance in UC patients has not been fully elucidated and remains debatable. Furthermore, appendix can play an important role in mucosal immune function in UC’s pathophysiology, considering previous studies on the inverse relationship between appendectomy and risk of UC development [14,15]. Most previous studies involved patients with distal UC [8,9,13]. To address this, we included distal and extensive UC patients. The present study aimed to evaluate the clinical features and the long-term outcomes of AOI, such as clinical significance and prognostic implications.

## 2. Materials and Methods

### 2.1. Patients

This study included UC patients who presented at Seoul St. Mary’s Hospital between January 2001 and December 2020. We retrospectively examined who were newly diagnosed or referred within 3 months of diagnosis. All patients fully examined medical and drug history. We only included patients who underwent index and follow-up colonoscopies. Incomplete colonoscopy that did not identify appendiceal orifice was excluded from index and follow-up colonoscopies. Patients with pan-colitis that continuously involved entire colon including cecal area and appendiceal orifice at initial diagnosis were excluded. This study was approved by the Institutional Review Board of Seoul St. Mary’s Hospital (approval number: KC21RASI0654) and was performed in accordance with the Declaration of Helsinki.

### 2.2. Study Design and Definitions

We analyzed the information regarding patients’ baseline characteristics, such as sex, age at diagnosis, date of UC diagnosis, mayo endoscopic subscore (MES) at diagnosis, presence of AOI, disease extent at diagnosis, presence of extraintestinal manifestation (EIM), and follow-up duration. Additionally, to evaluate long-term outcomes of AOI, we investigated complete ER, biologics use, hospitalization, and proximal disease extension on follow-up. AOI was defined as erythema, granularity, friability, and erosions or ulcerations at the appendiceal orifice. If AOI was confirmed in the index and follow-up colonoscopies, it was included as an AOI group. The disease extent of UC was defined according to the Montreal classification (E1, ulcerative proctitis; E2, left-sided UC; E3, extensive colitis). EIM was defined as eye or skin involvement, arthritis, and sclerosing cholangitis. Medication use was defined as use of systemic corticosteroids, immunomodulators (azathioprine, 6-mercaptopurine, cyclosporine, and methotrexate), or biologics (infliximab, adalimumab, vedolizumab, and golimumab). In Korea, a step-up approach is the mainstream treatment strategy, and more potent drugs, such as biologics, are administered to patients who are intolerant or refractory to first-line therapies. Complete ER was defined as MES 0. Hospitalization was defined as admission to manage disease flare-ups. Proximal disease extension was defined as the proximal extension of endoscopic mucosal inflammation beyond the initially involved segments (i.e., from E1 to E2 or E3, from E2 to E3). Patients were followed up regularly (usually every 1–3 months), based on their disease status and the physician’s discretion. All patients underwent sigmoidoscopy or colonoscopy if necessary (usually every 3–12 months) during follow-up period. Index colonoscopy was included those performed within 1 months after diagnosis. Follow-up colonoscopy was conducted regularly (usually every 12–36 months), based on their disease status and the physician’s discretion.

### 2.3. Statistical Analysis

Categorical data were analyzed using the chi-square test or Fisher’s exact test. Continuous data were compared using the Mann–Whitney U test. Values are reported as medians with interquartile ranges (IQRs). The cumulative risks of complete ER, use of biologics, hospitalization, and proximal disease extension were calculated using the Kaplan–Meier method, and the values were compared between groups using log-rank tests. Multivariate analysis was performed using the Cox proportional model to investigate the significant risk factors of the cumulative risks of complete ER. Variables first assessed by univariate analysis included: age, sex, AOI, disease extent, EIM, MES, systemic steroids, immunomodulator, and biologics. Predictors with *p* < 0.10 in univariate analysis were then evaluated in multivariate models. The hazard ratios (HRs) and 95% confidence intervals (CIs) were then calculated. *p*-values < 0.05 were considered statistically significant. All statistical analyses were performed using SPSS (version 21.0, Chicago, IL, USA).

## 3. Results

### 3.1. Baseline Characteristics of the Patients

Of the 318 UC patients enrolled, the AOI group included 140 patients (44.0%), and the non-AOI group included 178 patients (56.0%). The baseline characteristics of the patients are shown in Table 1. In the AOI group, the median age was 36 years (IQR, 24–50 years), 89 patients (63.6%) were male. The disease extent (E1, E2, E3) of UC at diagnosis was 60 patients (42.9%), 41 patients (29.3%), and 39 patients (27.9%) respectively. EIM was found in 9 patients (6.4%). One hundred fourteen patients (81.4%) showed MES 2 at initial diagnosis, and 26 patients (18.6%) MES3. All patients used 5-ASA agents. Use of systemic steroids, immunomodulators, and biologics during disease course were 85 patients (60.7%), 49 patients (35.0%), and 23 patients (16.4%), respectively. The median follow-up duration was 59 months (IQR, 34–96 months). The baseline characteristics, including age, sex, disease extent at diagnosis, EIM, MES at diagnosis, use of medication, and follow-up duration were not significantly different between the AOI and non-AOI groups.

### 3.2. Complete ER

**Figure 1:**
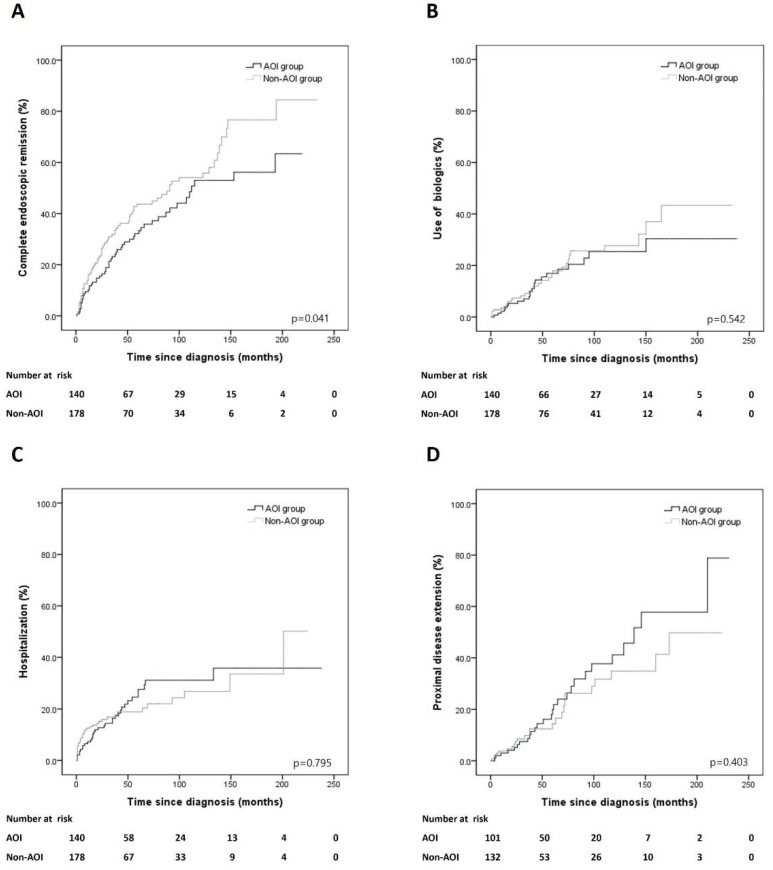
Cumulative risks of outcomes in patients with AOI and non-AOI groups. (**A**) Complete endoscopic remission, (**B**) use of biologics, (**C**) hospitalization, (**D**) proximal disease extension.

Complete ER was achieved in 53 (37.9%) patients with AOI and 80 (44.9%) patients without AOI. The cumulative risk of complete ER was different between the AOI and non-AOI groups (*p* = 0.041) (Figure 1A). In the multivariate Cox proportional analysis, AOI (HR, 0.656; 95% CI, 0.462–0.932; *p* = 0.019) and use of systemic steroids (HR, 0.556; 95% CI, 0.370–0.837; *p* = 0.005) were significantly associated with complete ER, respectively. No other risk factors were associated with complete ER (Table 2).

### 3.3. Use of Biologics

Biologics were required in 23 (16.4%) and 31 (17.4%) of patients between the AOI and non-AOI groups. The cumulative risk of use of biologics was not significantly different between the AOI and non-AOI groups (*p* = 0.542) (Figure 1B).

### 3.4. Hospitalization

Hospitalization was required in 33 (23.6%) and 36 (20.2%) patients between the AOI and non-AOI groups. The cumulative risk of hospitalization did not differ between the AOI and non-AOI groups (*p* = 0.795) (Figure 1C).

### 3.5. Proximal Disease Extension

Out of 101 and 132 distal UC patients between the AOI and non-AOI groups, proximal disease extension occurred in 27 (26.7%) and 24 (18.2%) patients, respectively. The cumulative risk of proximal disease extension was not different between the AOI and non-AOI groups (*p* = 0.403) (Figure 1D).

## 4. Discussion

This study compared the long-term outcomes of UC patients with and without AOI. Although there is no consensus on its clinical significance, AOI is considered to be a skip lesion of UC, and not a result of improvement due to medical treatment. It is more frequently found in patients with distal UC [5,16]. The prevalence of AOI is not low, ranging from approximately 7.9–75% of all UC patients, including newly diagnosed and pre-existing cases [9,10,11,12]. We found that AOI was endoscopically present in 46.5% of UC patients, of which 60% were AOI-positive at diagnosis, and 40% showed positive conversion during follow-up. The most notable finding of our study was that the cumulative risk of complete ER differed significantly between AOI and non-AOI groups (*p* = 0.041). Moreover, multivariate Cox proportional analysis also showed a significant relationship between AOI and complete ER (*p* = 0.019). The reason for this difference is unclear. Further studies are needed to validate these findings. ER is associated with a lower risk of clinical relapse, use of immunomodulators, hospitalization, colectomy, UC-related dysplasia, and colorectal cancer [3,4,17,18,19]. Endoscopic mucosal inflammatory activity is negatively correlated with the patients’ quality of life [20,21]. Patients who achieved ER were associated with better long-term outcomes than those who achieved clinical remission alone [22,23] Furthermore, ER is a more objective parameter for evaluating UC activity than clinical remission. Therefore, ER has been regarded as an important treatment goal in UC [3,24,25]. In our study, complete ER was defined as MES 0. Several studies reported that MES 0 (complete ER) has a lower relapse rate than MES 1 (partial ER) in UC patients [22,23,26,27]. Furthermore, recent studies reported that MES 0 was significantly associated with histological healing compared to MES 1 [28,29,30]. Recently, interest in histological healing as a treatment goal in patients with UC is increasing, and histological inflammation has been associated with a higher incidence of clinical relapse and development of colorectal neoplasia [27,30,31]. Considering this, the results of our study may have important implications for the treatment of UC patients with AOI in the future. To the best of our knowledge, this is the first study to find an association between AOI and lower rate of complete ER. Previous studies were conducted on AOI and UC remission [9,11]. Byeon et al. [9] reported that AOI was not associated with remission. They adopted clinical criteria to assess disease remission, which was defined as symptom improvement that resulted in no rectal bleeding and a bowel frequency of <3 times per day. They included only newly diagnosed patients with distal UC who were initially AOI-positive. Matsumoto et al. [11] reported that AOI in distal UC patients was associated with ER within a 12-month follow-up period. Unlike our study, ER was defined as an endoscopically distorted mucosal vascular pattern (MES 1). They included patients with pre-existing or newly diagnosed distal UC. No data on complete ER in distal and extensive UC patients with AOI have been reported. Our study provides evidence that can be used to determine further treatment strategies for complete ER in UC patients with AOI. Our study has several strengths. First, unlike previous studies conducted before the use of biologics began in earnest, our study included the use of biologics. The introduction of biologics has greatly changed the treatment paradigm of patients with UC and has significantly altered the disease outcome. Second, we included only patients who were newly diagnosed or referred patients within 3 months after diagnosis and who underwent index and follow-up colonoscopies. Nevertheless, this study included a relatively large number of patients. Third, we investigated the clinical significance of AOI in UC by including all findings of AOI at the index and follow-up colonoscopies. Moreover, we included all UC patients, including those with distal and extensive UC However, our study has several limitations. First, this was a retrospective, single-center study; hence, there may have been inherent bias. Second, we cannot exclude the possibility that some cases of positive conversion of AOI were missed during the follow-up period. In UC patients, there is a possibility that a positive conversion of AOI may be missed because in many cases, sigmoidoscopy is performed instead of a full colonoscopy depending on the extent of the disease during the follow-up period. Third, not all UC patients underwent colonoscopy in real clinical practice because it is not easy to complete a full colonoscopy during the acute phase. This might have been associated with selection bias.

## 5. Conclusions

AOI was significant associated with a lower rate of complete ER in UC patients. In clinical practice, UC patients achieving complete ER have a substantially lower risk of relapse. Therefore, a meticulous treatment strategy may be recommended for complete ER in UC patients with AOI. Future well-designed large-scale studies are needed to ensure our results.

## Figures and Tables

**Table 1:** Baseline characteristics according to appendiceal orifice inflammation.

	Total	AOI Group	Non-AOI Group	*p*
	(*n* = 318)	(*n* = 140)	(*n* = 178)	
Age, years (median, IQR)	36 (25–51)	36 (24–50)	36 (26–51)	0.802
Age, Montreal classification (*n*, %)				0.952
A1	9 (2.8)	4 (2.9)	5 (2.8)	
A2	174 (54.7)	78 (55.7)	96 (53.9)	
A3	135 (42.5)	58 (41.4)	77 (43.3)	
Sex, male (n, %)	192 (60.4)	89(63.6)	103 (57.9)	0.302
Disease extent, at diagnosis (*n*, %)				0.809
E1	134 (42.2)	60 (42.9)	74 (41.6)	
E2	99 (31.1)	41 (29.3)	58 (32.6)	
E3	85 (26.7)	39 (27.9)	46 (25.8)	
EIM (*n*, %)	23 (7.2)	9 (6.4)	14 (7.9)	0.623
MES, at diagnosis (*n*, %)				0.464
2	253 (79.6)	114 (81.4)	139 (78.1)	
3	65 (20.4)	26 (18.6)	39 (21.9)	
Use of medications (*n*, %)				
Systemic steroids	202 (63.5)	85 (60.7)	117 (65.7)	0.356
Immunomodulators	116 (36.5)	49 (35.0)	67 (37.6)	0.627
Biologics	54 (17.0)	23 (16.4)	31 (17.4)	0.816
Follow-up duration, months (median, IQR)	52 (26–102)	59 (34–96)	50 (23–111)	0.105

AOI, appendiceal orifice inflammation; IQR, interquartile range; EIM, extraintestinal manifestation; MES, Mayo endoscopic subscore.

**Table 2:** Cox proportional hazards model for clinical factors associated with complete endoscopic remission.

	Univariate		Multivariate	
	HR (95% CI)	*p*	HR (95% CI)	*p*
Age, Montreal Classification			-	-
A1	Reference			
A2	1.320 (0.414–4.206)	0.638		
A3	1.326 (0.415–4.243)	0.634		
Sex			-	-
Male	Reference			
Female	0.805 (0.565–1.145)	0.805		
AOI				
No	Reference		Reference	
Yes	0.698 (0.492–0.989)	0.043	0.656 (0.462–0.932)	0.019
Disease extent, at diagnosis			-	-
E1	Reference			
E2	0.826 (0.552–1.234)	0.35		
E3	0.855 (0.560–1.307)	0.47		
EIM			-	-
No	Reference			
Yes	0.861 (0.473–1.570)	0.626		
MES, at diagnosis			-	-
2	Reference			
3	0.807 (0.525–1.241)	0.33		
Use of medications				
Systemic steroids	0.559 (0.394–0.794)	0.001	0.556 (0.370–0.837)	0.005
Immunomodulators	0.720 (0.502–1.032)	0.073	0.919 (0.606–1.395)	0.692
Biologics	0.852 (0.547–1.328)	0.48	-	-

HR, hazard ratio; CI, confidence interval; AOI, appendiceal orifice inflammation; IQR, interquartile range; EIM, extraintestinal manifestation; MES, Mayo endoscopic subscore.

## Data Availability

Not applicable.
